# A statewide investigation of geographic lung cancer incidence patterns and radon exposure in a low-smoking population

**DOI:** 10.1186/s12885-018-4002-9

**Published:** 2018-01-31

**Authors:** Judy Y. Ou, Brynn Fowler, Qian Ding, Anne C. Kirchhoff, Lisa Pappas, Kenneth Boucher, Wallace Akerley, Yelena Wu, Kimberly Kaphingst, Garrett Harding, Deanna Kepka

**Affiliations:** 1 Huntsman Cancer Institute at the University of Utah, 2000 Circle of Hope Drive, Salt Lake City, UT 84112 USA; 2 Department of Pediatrics, University of Utah, 295 Chipeta Way, Salt Lake City, UT 84112 USA; 3 Study Design and Biostatistics Center, University of Utah, 295 Chipeta Way, Salt Lake City, UT 84112 USA; 4 Department of Family and Preventive Medicine, University of Utah, 375 Chipeta Way, Salt Lake City, UT 84112 USA; 5 Department of Communication, University of Utah, 255 S Central Campus Dr., Rm 2400, Salt Lake City, UT 84112 USA; 6 College of Nursing, University of Utah, 10 South 2000 East, Salt Lake City, UT 84112 USA

**Keywords:** Lung cancer, Radon, Public health, Geography, Non-genetic risk factors

## Abstract

**Background:**

Lung cancer is the leading cause of cancer-related mortality in Utah despite having the nation’s lowest smoking rate. Radon exposure and differences in lung cancer incidence between nonmetropolitan and metropolitan areas may explain this phenomenon. We compared smoking-adjusted lung cancer incidence rates between nonmetropolitan and metropolitan counties by predicted indoor radon level, sex, and cancer stage. We also compared lung cancer incidence by county classification between Utah and all SEER sites.

**Methods:**

SEER*Stat provided annual age-adjusted rates per 100,000 from 1991 to 2010 for each Utah county and all other SEER sites. County classification, stage, and sex were obtained from SEER*Stat. Smoking was obtained from Environmental Public Health Tracking estimates by Ortega et al. EPA provided low (< 2 pCi/L), moderate (2–4 pCi/L), and high (> 4 pCi/L) indoor radon levels for each county. Poisson models calculated overall, cancer stage, and sex-specific rates and *p*-values for smoking-adjusted and unadjusted models. LOESS smoothed trend lines compared incidence rates between Utah and all SEER sites by county classification.

**Results:**

All metropolitan counties had moderate radon levels; 12 (63%) of the 19 nonmetropolitan counties had moderate predicted radon levels and 7 (37%) had high predicted radon levels. Lung cancer incidence rates were higher in nonmetropolitan counties than metropolitan counties (34.8 vs 29.7 per 100,000, respectively). Incidence of distant stage cancers was significantly higher in nonmetropolitan counties after controlling for smoking (16.7 vs 15.4, *p* = 0.02*). Incidence rates in metropolitan, moderate radon and nonmetropolitan, moderate radon counties were similar. Nonmetropolitan, high radon counties had a significantly higher incidence of lung cancer compared to nonmetropolitan, moderate radon counties after adjustment for smoking (41.7 vs 29.2, *p* < 0.0001*). Lung cancer incidence patterns in Utah were opposite of metropolitan/nonmetropolitan trends in other SEER sites.

**Conclusion:**

Lung cancer incidence and distant stage incidence rates were consistently higher in nonmetropolitan Utah counties than metropolitan counties, suggesting that limited access to preventative screenings may play a role in this disparity. Smoking-adjusted incidence rates in nonmetropolitan, high radon counties were significantly higher than moderate radon counties, suggesting that radon was also major contributor to lung cancer in these regions. National studies should account for geographic and environmental factors when examining nonmetropolitan/metropolitan differences in lung cancer.

## Background

Lung cancer is the leading cause of cancer-related mortality in the state of Utah despite having the lowest smoking prevalence and lung cancer incidence rate in the United States [1, 2]. Second only to tobacco, radon is a prominent risk factor for lung cancer in nonsmokers [3], contributing to an estimated 20,000 lung cancer deaths in the United States yearly [4, 5]. Pooled analyses in Europe and North America supports residential radon as a significant contributor to lung cancer in smokers and nonsmokers [3, 6–9], although absolute and relative effects differ by smoking status [10]. Radon, a ubiquitous naturally occurring radioactive gas formed by the decay of uranium, enters homes and is inhaled by occupants. Chronic inhalation leads to lung cancer in a dose dependent fashion, but mitigation can reduce exposure and lung cancer risk. It affects both smokers and non-smokers, but effects are particularly adverse for smokers [11]. Since only 9% of adults in Utah are current cigarette smokers, and the state has a potential for high radon emission due to the soil’s uranium content [12, 13], radon may contribute to a larger portion of Utah’s lung cancer burden than other regions in the United States. In Utah, 30% of homes have indoor radon levels of 4 picocuries per liter (pCi/L) or higher (4 pCi/l = 148 Bg/m^3^) [14], which is defined as hazardous to human health [15]. Nationwide, 7% of homes have similar radon levels [16].

Lung cancer mortality in Utah may also be affected by the geographic distribution of its population. Twenty percent of Utah’s population live in nonmetropolitan or frontier regions with limited access to clinics providing preventative care and cancer treatment [17]. Studies examining lung cancer incidence patterns within the United States vary greatly in their conclusions about the role of variation in geographic area on lung cancer incidence [18–21]. Some large studies in the United States report higher incidence and mortality rates of lung cancer in nonmetropolitan regions than metropolitan regions [18, 19, 22]. Other studies found metropolitan areas typically report lower lung cancer mortality rates and higher incidence of late state lung cancer than nonmetropolitan region [21], which may be attributed to the availability of diagnostic and treatment facilities. A major explanation for these differences could be the increased prevalence of smoking in nonmetropolitan regions [18], as well as personal risk factors of lower educational attainment and lower income [18, 20]. To our knowledge, few analyses examine nonmetropolitan and metropolitan differences in lung cancer incidence while including the contextual factor of radon exposure. Since smoking contributes to an overwhelming number of lung cancer cases compared to radon, studying lung cancer incidence rates in a low-smoking population would provide more information about the total burden of lung cancer cases attributed to radon in these populations.

Hazardous levels of radon and lack of health care resources in nonmetropolitan Utah pose two major challenges to preventing lung cancer and lung cancer mortality in this low smoking population. This paper describes the population of metropolitan and nonmetropolitan Utah counties, and compares lung cancer incidence rates among Utah counties from 1991 to 2010 by metropolitan classification and radon level. We also examine patterns in lung cancer incidence by metropolitan and nonmetropolitan counties between Utah and the United States.

## Methods

The Surveillance, Epidemiology, and End Results Program (SEER) houses the statistical software program SEER*Stat [23, 24]. SEER*Stat contains counts of lung cancer cases diagnosed between 1991 and 2010 in seven sites across the United States including Utah, cancer stage and sex of the patient, and population attributes of income, education, employment, and poverty by county. SEER*Stat also contains the population, population years, and 2000 United States standard population [25].

SEER*Stat includes metropolitan or nonmetropolitan county classifications based on the Nonmetropolitan-Metropolitan Continuum Code Definitions [26], which integrates population density, urbanization, and daily commuting patterns based on census tracts to define metropolitan and nonmetropolitan counties. We classified counties with RUCA codes between 1 and 3 as metropolitan, and considered counties with RUCA codes between 4 and 9, which included small towns, nonmetropolitan, and micropolitan areas, as nonmetropolitan [27].

Predicted average indoor residential radon levels for each county were abstracted from the Environmental Protection Agency from 1991 to 2010 [28]. Low radon levels were defined as levels of 2 pCi/L or less; moderate levels were between 2 and 4 pCi/L (74 to 148 Bg/m^3^); high levels were greater than 4 pCi/L [15].

We also obtained the estimated smoking prevalence by county from 1991 to 2010 in 4 year intervals from Ortega et al. [29]. The smoking estimates were consistent with smoking estimates from Utah’s 2010 Behavioral Risk Factor Surveillance System survey.

### Statistical methods

Annual counts and incidence rates per 100,000 for each Utah county were generated using SEER*Stat software. Rates from 1991 to 2010 were adjusted to the 2000 United States population distribution in 19 age groups [25]. We also obtained annual rates by county classification for all other SEER sites in the United States. Data from SEER 9 was used for all calculations.

We summarized county-level population characteristics, including education, age, income, race/ethnicity, employment, and income as reported by SEER*Stat. Multivariate Poisson regression models compared the age-adjusted lung cancer incidence rates between metropolitan and nonmetropolitan counties; analyses adjusting for smoking were also run. Among non-metropolitan counties, we compared age-adjusted incidence rates, and age- and smoking-adjusted rates between counties with high and moderate radon levels. Gender and stage specific lung cancer incidence rates were compared by county classification and radon level. We obtained *p*-values for models with and without adjustment for smoking.

Annual lung cancer incidence rates and 95% confidence limits for Utah and all other SEER sites across the United States were plotted using LOESS curves, a nonparametric local regression smoothing method. The lung cancer incidence rates were compared by metropolitan classification between Utah and all other SEER sites.

SAS 9.4 was used for all analyses. Significance was defined by two sided *p*-values less than 0.05.

## Results

Of the 29 counties in Utah, 10 were classified as metropolitan counties and 19 as nonmetropolitan counties. As expected, the population in metropolitan counties was larger than nonmetropolitan counties for all years. In 2010, 2,459,621 people lived in metropolitan counties and 315,472 people lived nonmetropolitan counties. No counties in Utah had low radon levels. All metropolitan counties had moderate radon levels. Twelve nonmetropolitan counties (63%) had moderate radon levels; seven (37%) had high radon levels.

Population attributes of Utah residents differed by metropolitan classification and radon level (Table 1). Compared to metropolitan counties, nonmetropolitan counties had a higher percent of residents with a high school degree or less (Nonmetropolitan = 14.3%, Metropolitan = 11.0%), fewer residents with a college degree (Nonmetropolitan = 16.8%, Metropolitan = 25.4%), lower median incomes (Nonmetropolitan = $34.3 K, Metropolitan = $45.8 K), and a higher percent of persons below the federal poverty level (Nonmetropolitan = 12%, Metropolitan = 6.7%). The prevalence of smoking was higher in all nonmetropolitan counties (15.6–17.5%) than metropolitan counties (12.8–14.3) from 1991 to 2010 (Table 1).

**Table 1:** Attributes of Populations living in Metropolitan and Nonmetropolitan Utah Counties

	Metropolitan		Nonmetropolitan					
	Moderate Radon *n* = 10		All Nonmetropolitan *n* = 19		Moderate Radon *n* = 12		High Radon *n* = 7	
	Median %	Range	Median %	Range	Median %	Range	Median %	Range
Education								
High school graduate or less	11.0	7.4–17.1	14.3	8.5–30.4	13.4	8.5–30.4	17.5	14.2–20.2
Bachelor’s degree or more	25.4	12.2–45.5	16.8	11.6–26.3	19.9	11.6–26.3	14.5	12.3–22.9
Age								
18 years or less	32.7	29.8–38.6	33.5	23.2–39.3	33.9	23.2–39.3	33.2	26.9–36.8
65 years or more	7.7	4.9–17.0	12.5	8.4–17.1	12.9	8.4–16.7	12.5	9.4–17.1
Income								
Median household income (thousands)	45.8	37.2–65.0	34.3	28.1–49.6	34.9	28.1–49.6	33.0	29.6–35.8
Persons below poverty	6.7	5.1–15.4	12.0	5.5–31.4	10.7	5.5–31.4	14.8	10.8–16.8
Employment								
Unemployed	4.7	2.8–6.0	6.4	2.2–15.1	5.3	2.2–15.1	7.7	6.3–8.9
Race/Ethnicity								
Non-white residents (total)	3.4	0.4–6.7	2.3	0.5–56.5	2.1	0.5–56.5	2.9	1.6–10.1
Black	0.5	0.2–1.7	0.2	0.1–1.2	0.2	0.05–1.2	0.3	0.1–0.5
American Indian/Alaska Native	0.8	0.1–2.0	1.3	0–56.1	1.0	0–56.1	2.2	1.1–9.7
Asian/Pacific Islander	1.4	0.1–4.3	0.4	0–1.2	0.4	0–1.2	0.3	0.2–1.0
Hispanic	6.7	1.4–12.8	4.5	1.8–10.3	4.6	1.8–7.2	4.5	2.6–10.3
Other minority	9.7	1.7–18.1	7.3	2.3–59.5	6.5	2.3–59.5	9.4	5.2–13.2
Smoking Prevalence								
1991–1995	14.3	8.0–25.4	17.9	12.2–28.3	17.5	12.2–28.3	21.8	14.5–25.4
1996–2000	13.8	7.7–24.4	17.2	11.8–27.2	16.9	11.8–27.2	19.7	13.9–24.5
2001–2005	13.3	7.4–23.5	16.5	11.3–26.1	16.2	11.3–26.1	18.9	13.4–23.5
2006–2010	12.8	7.1–22.6	15.9	10.9–25.1	15.6	10.9–25.1	18.2	12.9–22.6

Attributes were collected at the county-level. The median percent represents the median of the aggregate percentage of individuals with that characteristic by county. Moderate radon: 2–4 pCi/L (74 to 148 Bg/m3). High radon: > 4 pCi/L (> 148 Bg/m3)

When population attributes were examined by radon and metropolitan classification, nonmetropolitan counties with high radon levels had the highest percent of residents with a high school degree or less (Nonmetropolitan, high radon = 17.5%; Nonmetropolitan, moderate radon = 13.4%; Metropolitan, moderate radon = 11.0%), the highest percent of persons below the federal poverty level (Nonmetropolitan, high radon = 14.8%; Nonmetropolitan, moderate radon = 10.7%; Metropolitan, moderate radon = 6.7%), and the highest unemployment rates (Nonmetropolitan, high radon, =7.7%; Nonmetropolitan, moderate radon = 5.3%; Metropolitan, moderate radon = 4.7%). Nonmetropolitan, high radon counties had the highest smoking prevalence for all years of the study.

Age-adjusted lung cancer incidence rates per 100,000 for metropolitan and nonmetropolitan counties also differed (Table 2) in the non-smoking adjusted model (Model 1 *p*-values) and the smoking-adjusted model (Model 2 p-values). Prior to adjustment for smoking, lung cancer incidence rates in Table 2, Model 1 in nonmetropolitan counties were significantly higher than in metropolitan counties (Nonmetropolitan = 34.8; Metropolitan = 29.7, *p* < 0.01), but not significant in the smoking adjusted model (*p* = 0.19). Rates for all cancer stages, and among men and women in nonmetropolitan counties were significantly higher than metropolitan regions in the smoking-unadjusted model. Only distant (Metropolitan = 15.4; Nonmetropolitan = 16.7, *p* = 0.02) and unstaged cancer stages (Metropolitan = 2.7; Nonmetropolitan = 4.9, *p* < 0.001) remained significant after the inclusion of smoking.

**Table 2:** Comparison of Age-Adjusted Lung Cancer Incidence Rates per 100,000 from 1991 to 2010 in Metropolitan and Nonmetropolitan Utah Counties by Stage and Sex

	Metropolitan			Nonmetropolitan			Model 1: Not adjusted for smoking	Model 2: Adjusted for smoking
	Rate	Count	Person-years	Rate	Count	Person-years	*P*-value	*P*-value
Overall incidence	29.7	8302	40,106,205	34.8	1630	5,297,867	<.0001*	0.19
Stage								
Localized	5.2	1462	40,106,205	6.0	285	5,297,867	0.02*	0.83
Regional	6.3	1792	40,106,205	7.2	336	5,297,867	0.04*	0.15
Distant	15.4	4317	40,106,205	16.7	784	5,297,867	0.02*	0.02*
Unstaged	2.7	731	40,106,205	4.9	225	5,297,867	< 0.0001*	< 0.0001*
Sex								
Male	39.5	4963	20,073,510	45.7	1004	2,662,232	< 0.0001*	0.24
Female	21.9	3339	20,032,695	25.4	626	2,635,635	0.0003*	0.46

Rates adjusted to the 2000 United States standard population. Person-years shown here are not age-adjusted. *Indicate *p*-values < 0.05 are significant

Among nonmetropolitan counties, high radon counties had a higher lung cancer incidence rate than moderate radon counties (Table 3), even after adjusting for smoking (Moderate radon = 29.2; High radon = 41.7, *p* < 0.0001*). Prior to adjustment for smoking, incidence rates for every cancer stage were significantly higher in high radon counties than moderate radon counties. With the exception of localized stage lung cancer, incidence of regional and distant cancer stages remained significant after adjustment for smoking. Males living in high radon counties also had significantly higher rates of lung cancer than males living in moderate radon counties, for both the smoking-unadjusted (High radon = 57.3; Moderate radon = 36.3, p < 0.0001*) and smoking-adjusted models (p < 0.0001*). When comparing rates between moderate radon, metropolitan counties in Table 2 with the moderate radon, nonmetropolitan counties in Table 3, we found that the rates were very similar for overall incidence (Metropolitan, moderate radon =29.7; Nonmetropolitan, moderate radon = 29.2), cancer stage, and sex.

**Table 3:** Comparison of Age-Adjusted Lung Cancer Incidence Rates per 100,000 from 1991 to 2010 by Radon Level in Nonmetropolitan Utah Counties

	Nonmetropolitan Counties: Moderate Radon			Nonmetropolitan Counties: High Radon			Model 1: Not adjusted for smoking	Model 2: Adjusted for smoking
	Rate	Count	Person-years	Rate	Count	Person-years	*P*-value	*P*-value
Overall incidence	29.2	760	3,040,765	41.7	870	2,257,102	< 0.0001*	< 0.0001*
Stage								
Localized	5.3	139	3,040,765	6.9	146	2,257,102	0.02*	0.09
Regional	5.7	149	3,040,765	9.0	187	2,257,102	< 0.0001*	0.0008*
Distant	14.3	370	3,040,765	19.8	414	2,257,102	< 0.0001*	0.0008*
Unstaged	4.0	102	3,040,765	6.0	123	2,257,102	0.003*	0.02*
Sex								
Male	36.3	446	1,530,326	57.3	558	1,131,906	< 0.0001*	< 0.0001*
Female	23.0	314	1,510,439	28.4	312	1,125,196	0.007*	0.11

Rates are adjusted to the 2000 United States standard population. Person-years shown here are not age-adjusted. *Indicate *p*-values <0.05 are significant. Moderate radon: 2–4 pCi/L (74 to 148 Bg/m3). High radon: > 4 pCi/L (> 148 Bg/m3)

**Fig. 1:**
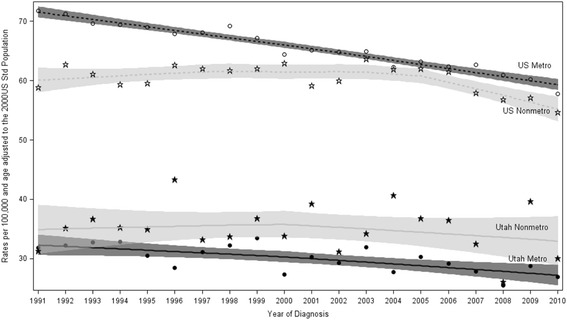
LOESS Smoothed Line and 95% Confidence Limits for Annual Age-Adjusted Lung Cancer Incidence Rates from 1991 to 2010 for Utah and all SEER sites in the United States by County Classification. US Metro: Metropolitan counties in all other SEER site. US Nonmetro: Nonmetropolitan counties in all other SEER sites. Utah Metro: Metropolitan counties in Utah. Utah Nonmetro: Nonmetropolitan counties in Utah

Age-adjusted lung cancer rates also differed for all other SEER sites compared to Utah by metropolitan region (Fig. 1). The lung cancer incidence rate in Utah was much lower than other SEER sites for all years studied. Within all other SEER sites, metropolitan regions had a higher lung cancer incidence rate than nonmetropolitan regions. In contrast, incidence rates in nonmetropolitan regions of Utah were consistently higher than metropolitan regions for all years, with significantly higher rates observed in nonmetropolitan regions from 1997 to 2007.

## Discussion

Lung cancer is the second most commonly diagnosed cancer in the United States and the leading cause of cancer-related death [30, 31], contributing to an estimated 158,080 deaths in 2016 alone [32]. Although many studies show significant variation in nonmetropolitan-metropolitan differences in lung cancer incidence on a national scale [19], we report a consistent higher incidence of lung cancer in nonmetropolitan Utah counties than metropolitan counties. This pattern is opposite the trend seen in other SEER sites across the United States, where lung cancer incidence is higher in metropolitan regions than nonmetropolitan regions. After controlling for smoking, we find that nonmetropolitan areas in Utah have a significantly higher rate of distant lung cancers, suggesting that nonmetropolitan region of residence is a contributing factor to late stage cancer at diagnosis and may influence mortality.

All metropolitan Utah counties have predicted indoor radon levels between 2 and 4 pCi/L, but there is greater variability estimated for nonmetropolitan counties, which have some of the highest radon levels in the state. When we compare lung cancer rates in moderate radon regions, we see that the rate of lung cancer in nonmetropolitan, moderate radon and metropolitan, moderate radon counties is nearly identical. In contrast, overall incidence in high radon, nonmetropolitan counties is significantly higher than moderate radon, nonmetropolitan counties after adjustment for smoking. These results suggest that radon is a significant contributor to higher lung cancer incidence in high radon, nonmetropolitan Utah counties even after smoking is taking into consideration. Since the incidence rate among men is significantly higher in high radon counties than moderate radon counties after adjustment for smoking, radon may be a significant contributor to the burden of lung cancer among males residing in nonmetropolitan counties.

Utah has the lowest smoking prevalence and the lowest number of lung cancer cases attributed to smoking than any other state in the United States [33, 34], but we find that the prevalence of smoking in nonmetropolitan Utah counties from 2006 to 2010 of 18.2% is similar to the current national average of 15.1% [13]. The nonmetropolitan counties with the highest predicted indoor radon levels also have the highest smoking prevalence, meaning that residents of these counties may be at risk for dual exposure to radon and tobacco. In occupationally exposed populations, we find a higher absolute increase in risk per unit of radon in smokers than non-smokers, but larger relative risk per unit of radon in nonsmokers [10]. Although occupational radon exposure differs in dose and duration from residential radon exposure, these findings support a complex relationship between smoking status and radon that should be further explored in this low-smoking population. These regional differences in smoking prevalence should be taken into account when planning community-based interventions and epidemiologic research in geographically diverse states.

In addition to environmental risk factors for disease, populations in nonmetropolitan Utah counties display patterns of socioeconomic deprivation seen nationwide in similar nonmetropolitan regions [18, 20]. Utah’s nonmetropolitan counties consistently have a lower percent of residents with a bachelor’s degree and a higher percent of families living under the federal poverty level than metropolitan counties. Less education and poverty are risk factors for poorer cancer survival, and may impact nonmetropolitan populations differently than metropolitan populations [20]. In our study, the greatest social and economic disparities are seen between moderate radon, metropolitan counties and high radon, nonmetropolitan counties.

Since lung cancer prevention efforts largely focus on reducing smoking or other tobacco-use behaviors, additional funding should be dedicated towards reducing radon exposure as a significant risk factor. National educational efforts related to increasing awareness about radon and preventing exposure are limited. Out of the 65 state and territory cancer prevention plans created between 2005 and 2011, only 27, including Utah, mentioned activities related to radon and lung cancer prevention [35]. Utah’s 2016 to 2020 Comprehensive Cancer Prevention and Control Plan identifies radon as a priority, with an emphasis on testing homes for radon [16].

Despite these past efforts, awareness about the role of radon in lung cancer and the need for testing in Utah is low. Only 51.6% of the respondents in Utah’s 2013 Behavioral Risk Factor Surveillance System (BRFSS) could correctly identify lung cancer as a health risk of radon exposure, and only 20% of BRFSS respondents report testing their homes for radon [36]. When asked about reasons why they have not tested their home for radon, 34% say that they had not thought about it, 14% think they are not at risk, and 13% say that they do not know about radon [36]. These results demonstrate a need for radon awareness and mitigation activities directed towards the public.

This ecologic study is limited in the conclusions it can make about the dual roles of geographic area and radon in lung cancer in Utah. Ecologic studies are able to make group-level inferences that may not be true on the individual level [37]. However, we are able to identify county-level associations of radon and lung cancer in Utah. We are not able to examine how social and economic characteristics of the Utah population are associated with radon exposure and lung cancer incidence. We are not able to control for county-level air pollution, which is a potential confounder of the association between lung cancer and nonmetropolitan and metropolitan area of residence. Since the majority of traffic and other sources of pollution are in the metropolitan areas, we expect that air pollution will increase the rate of lung cancer cases in metropolitan counties, which is the opposite of what we report. Because of this, we do not expect air pollution to be a confounder in this study, but we will examine air pollution in future studies. We also have a relatively small number of counties (*n* = 19) since Utah is a relatively small state in terms of population. We are unable to adjust for calendar year, which may be an important confounder as it is a surrogate measure of lung cancer trends by county. Although we adjusted for smoking on the county-level, we may have some residual confounding from smoking from the study design and method of adjustment for smoking.

Despite these limitations, we are able to identify the age-adjusted lung cancer incidence rate for metropolitan and nonmetropolitan counties in Utah over a 20 year period, and examine differences in lung cancer incidence rates by radon level in nonmetropolitan counties. While county level estimates can be viewed as a weakness relative to individual or household exposure, a county-level assessment is still valuable in generating possible explanations for population trends in lung cancer incidence. Our study suggests that radon is a serious risk factor for lung cancer in the low smoking population of Utah, especially within nonmetropolitan counties.

Future research should assess patterns of radon testing, barriers to radon mitigation, and develop interventions and public health programs to improve awareness about radon and access to radon mitigation procedures. Previous interventions that provided tailored information and guidance to households and in primary care settings are effective at increasing awareness [38–40]. Differences in access to lung cancer screening and delays in treatment by geographic area, and the cumulative impact of smoking, radon exposure, and socioeconomic risk factors on lung cancer incidence and survival among nonmetropolitan residents in should be explored in future studies.

## Conclusions

Nonmetropolitan counties in Utah have a higher lung cancer incidence compared to metropolitan counties, which is opposite trends seen in other SEER sites. Incidence of lung cancer is similar between metropolitan and nonmetropolitan counties with similar radon levels, but significantly higher among high radon counties than moderate radon counties. Exposure to high levels of radon is the most likely explanation for the disparity in lung cancer incidence rates among nonmetropolitan Utah counties. Public health initiatives are needed to improve radon detection and facilitate radon mitigation activities to limit radon exposure among individuals in nonmetropolitan counties.

## Abbreviations

BRFSS: Behavioral Risk Factor Surveillance System
LOESS: Locally weighted scatterplot smoothing
pCi/L: Picocuries per liter
SEER: Surveillance, Epidemiology, and End Results
